# Epidemiological Characteristics of COVID-19 Resurgence in Areas Initially Under Control

**DOI:** 10.3389/fpubh.2021.749294

**Published:** 2021-11-23

**Authors:** Yazhen Li, Kai Yang, Shanshan Zha, Lingwei Wang, Rongchang Chen

**Affiliations:** Shenzhen Institute of Respiratory Diseases, Shenzhen People's Hospital (The Second Clinical Medical College, Jinan University; The First Affiliated Hospital, Southern University of Science and Technology), Guangdong, China

**Keywords:** COVID-19, epidemiological characteristics, infection route, preventive strategy, indigenous patient

## Abstract

**Objectives:** To investigate the epidemiological characteristics and infection routes of new cases in order to provide information for preventing COVID-19 resurgence in areas initially under control.

**Methods:** The information of new symptomatic and asymptomatic patients in Chinese mainland was collected. The location distribution, epidemic course, infection routes and patients' characteristics of outbreaks were described and analyzed.

**Results:** There were 43 new outbreaks with 3,795 symptomatic patients in Chinese mainland from March 21, 2020 to June 13, 2021. These outbreaks mainly occurred in central, border and coastal port cities. The main infection route of first generation indigenous patients was contact with imported cases and contaminated goods or environments. The infection routes of secondary generation patients mainly included family transmission, indoor social gathering infection, nosocomial infection and other infection routes. Family transmission was the most common infection route, and indoor social gathering was the most important reason for the large-scale outbreaks.

**Conclusions:** Strengthen the management of imported patients and staff in high-risk posts was the key point to avoid the first generation indigenous patients. Adequate family isolation, prompt management policies for indoor public place and monitor of population at risk of infection were key strategies for preventing COVID-19 resurgence in areas initially under control.

## Introduction

The coronavirus disease 2019 (COVID-19) pandemic, which began at the end of 2019, has been a worldwide epidemic of infectious disease (1). After effective prevention measures, the epidemic disease has been controlled and there are no new cases in a period of time in some countries (2). However, the COVID-19 pandemic is still prevalent in many countries worldwide (3). Over 170 million cases have been confirmed, and 70 thousand cases were newly diagnosed worldwide every day, which may lead to new epidemics in areas initially under control (4). The COVID-19 pandemic in Chinese mainland has been controlled in March 2020, and few new cases were confirmed for a long time (5). However, there were still 43 small-scale outbreaks caused by imported patients with COVID-19 by June 13, 2021, which lead to 3,795 new symptomatic patients. These new outbreaks revealed that there were still some gaps in the prevention of new pandemics in areas initially under control (6).

Therefore, to understand the epidemiological characteristics and main routes of the new outbreaks of COVID-19 in areas initially under control, we analyzed the epidemiological characteristics and infection routes of the outbreaks in different regions of Chinese mainland in recent 1 year, which will provide the basis for the corresponding preventive strategies.

## Materials and Methods

### Data Sources

The daily new indigenous symptomatic and asymptomatic patients, start date, duration, patient number and clinical subtype were collected from the official documents released by the Health Commission of the People's Republic of China and the provinces (7–18). The information of infection routes was extracted from the epidemiological investigation of the outbreaks in different cities from the Center for Disease Control and Prevention, as well as some public news. There were 43 new outbreaks in Chinese mainland during March 21, 2020 to June 13, 2021, in which 3,795 symptomatic patients were confirmed. Ten outbreaks with the largest number of symptomatic patients, including 3,290 patients (86.7% of all patients), were selected for further analysis (9–18).

### Definition of Indigenous Patients

The definitions of first and secondary generation indigenous patients were referred to guidelines for epidemiological investigation of COVID-19 release by the Chinese Center for Disease Control and Prevention (19). The definitions of symptomatic or asymptomatic patients were referred to the COVID-19 prevention and control program (8th edition) released by the National Health Commission of the People's Republic of China (20).

First generation indigenous patients were defined as the earliest symptomatic or asymptomatic patients in the epidemiological investigation of epidemics. According to the occupation of patients, the possible infection sources were investigated, including imported cases, imported goods or related environment contaminated by severe acute respiratory syndrome coronavirus 2 (SARS-CoV-2).

Second generation indigenous patients were defined as symptomatic or asymptomatic patients contacted with the first generation indigenous patients within 14 days without other potential sources of infection. After identifying the first generation patients, the infection routes of the second generation patients were determined by investigating the contacts with the first generation patients, and were classified into family transmission, indoor social gathering infection (dinner party, wedding banquet, workplace, training class, market, etc.), nosocomial infection and other infection routes. The definitions of other secondary generation of patients were similar.

### Epidemiological Analysis

The number of symptomatic and asymptomatic patients, start date, duration and clinical subtype of patients in different epidemics were summarized in the descriptive epidemiological analysis. The numbers of daily symptomatic patients were used to describe the epidemic curves of different outbreaks. Statistical map was used to describe the location distribution of outbreaks, in which the area of the circle was proportional to the number of patients. The infection routes of first and secondary generation patients were extracted and summarized from the epidemiological investigation to identify the key infection routes of patients.

## Results

### The Courses of New Outbreaks in Chinese Mainland

**Figure 1:**
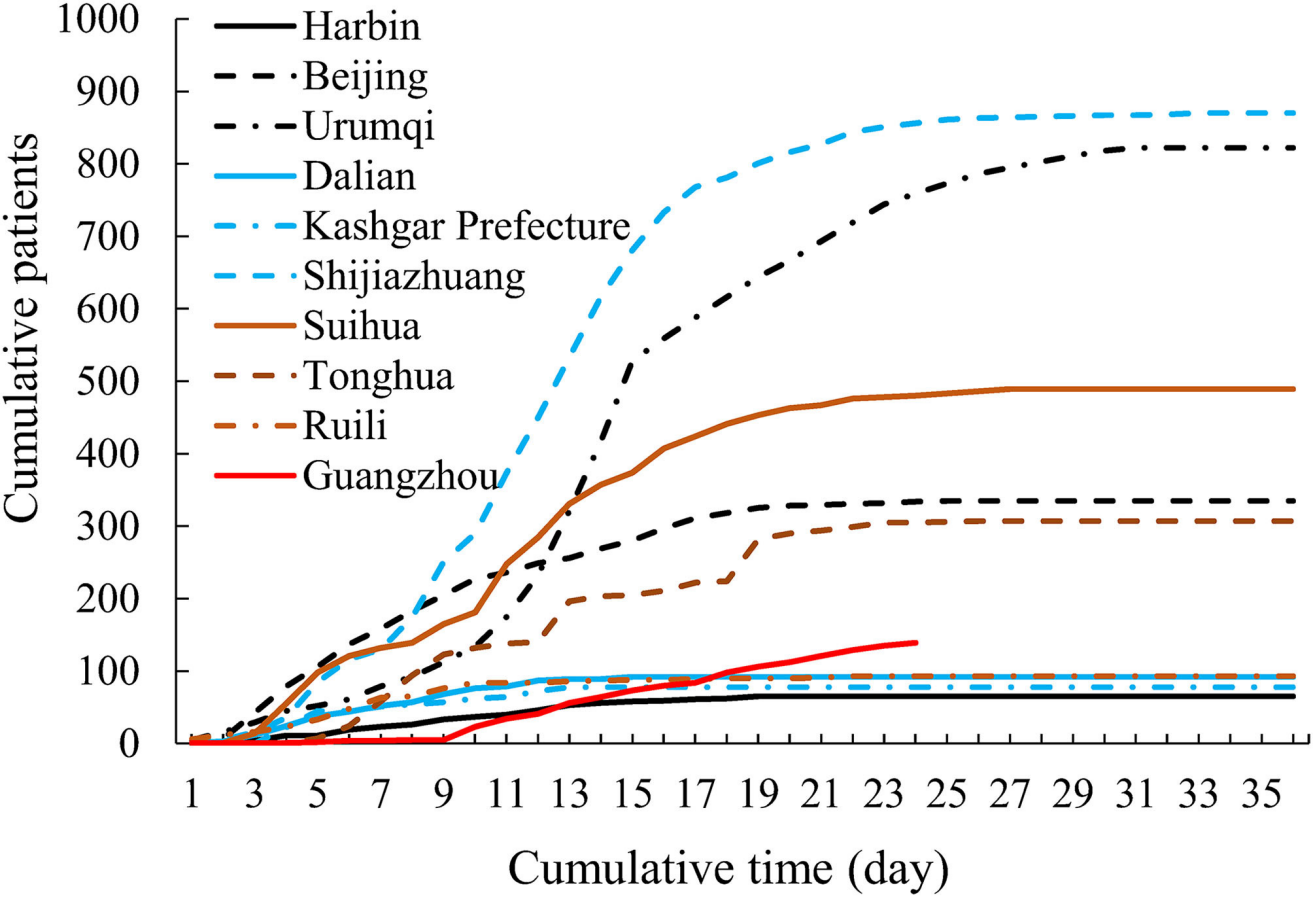
The epidemic curve of new indigenous outbreaks in 10 cities with the largest number of patients in Chinese mainland.

On March 21, 2020, the first indigenous patient cause by imported patient was reported in Guangzhou, who did not cause further transmission. However, in the past year, there have been some new outbreaks with a large number of indigenous confirmed cases in Harbin, Beijing, Urumqi, Dalian and other cities (Figure 1). In April 2020, an imported patient, who returned to Harbin from America, leaded to an epidemic of 65 symptomatic and 31 asymptomatic patients by contaminated environments, dinner party and nosocomial infection before diagnosis (Table 1). In June and July 2020, there were 335 symptomatic and 31 asymptomatic patients in Beijing, and 92 symptomatic and 97 asymptomatic patients in Dalian, respectively. The source of these two outbreaks was probably the imported cold chain food, and the large-scale outbreaks were caused by family transmission and indoor social gathering. The new indigenous outbreaks also outbroke in other cities between July 2020 and May 2021 (Table 1). Although the infection sources were not yet determined, the outbreaks were all caused by large-scale indoor gatherings (wedding banquet, training class, etc.).

**Table 1:** Basic information of patients in different outbreaks.

**City**	Start date^*^	**Duration (day)^#^**	**Patients number**	**Clinical subtype (severe/moderate/mild)**	**Asymptomatic patients (all/symptomatic later)**
Harbin	April 9, 2020	19	65	-	31/9
Beijing	June 11, 2020	25	335	6/274/55	31/-
Urumqi	July 16, 2020	33	822	-	387/154
Dalian	July 22, 2020	15	92	1/68/23	97/72
Kashgar Prefecture	October 24, 2020	15	78	-	423/77
Shijiazhuang	January 2, 2021	36	870	-	366/201
Suihua	January 9, 2021	27	489	-	547/139
Tonghua	January 12, 2021	29	307	29/128/332	263/252
Ruili	March 30, 2021	22	93	0/53/40	44/19
Guangzhou^Δ^	May 21, 2021	-	139	-	42/34

* *The start date was the day when the first symptomatic patient or asymptomatic patient was reported by the Health Commission*.

# *The duration included the time of first and last asymptomatic patient*.

Δ *By June 13, 2021*.

In May, four outbreaks also occurred in Yingkou, Lu'an, Guangzhou and Shenzhen. Among them, the outbreaks in Yingkou and Lu'an belonged to the same transmission chain, and the source might be cold chain food in Yingkou, resulting in 24 symptomatic and 23 asymptomatic patients in both cities. On May 21, a new indigenous patient was reported in Guangzhou, which leaded to rapid spread of COVID-19 through family transmission and indoor gathering, resulting in fifth generation of transmission and 139 symptomatic cases. On the same day, one indigenous asymptomatic patients was reported in Shenzhen, and four symptomatic and 12 asymptomatic patients were reported in the next few days, who were all employees of an international freighter operation company and their close contacts.

### Location Distribution of New Indigenous Outbreaks

**Figure 2:**
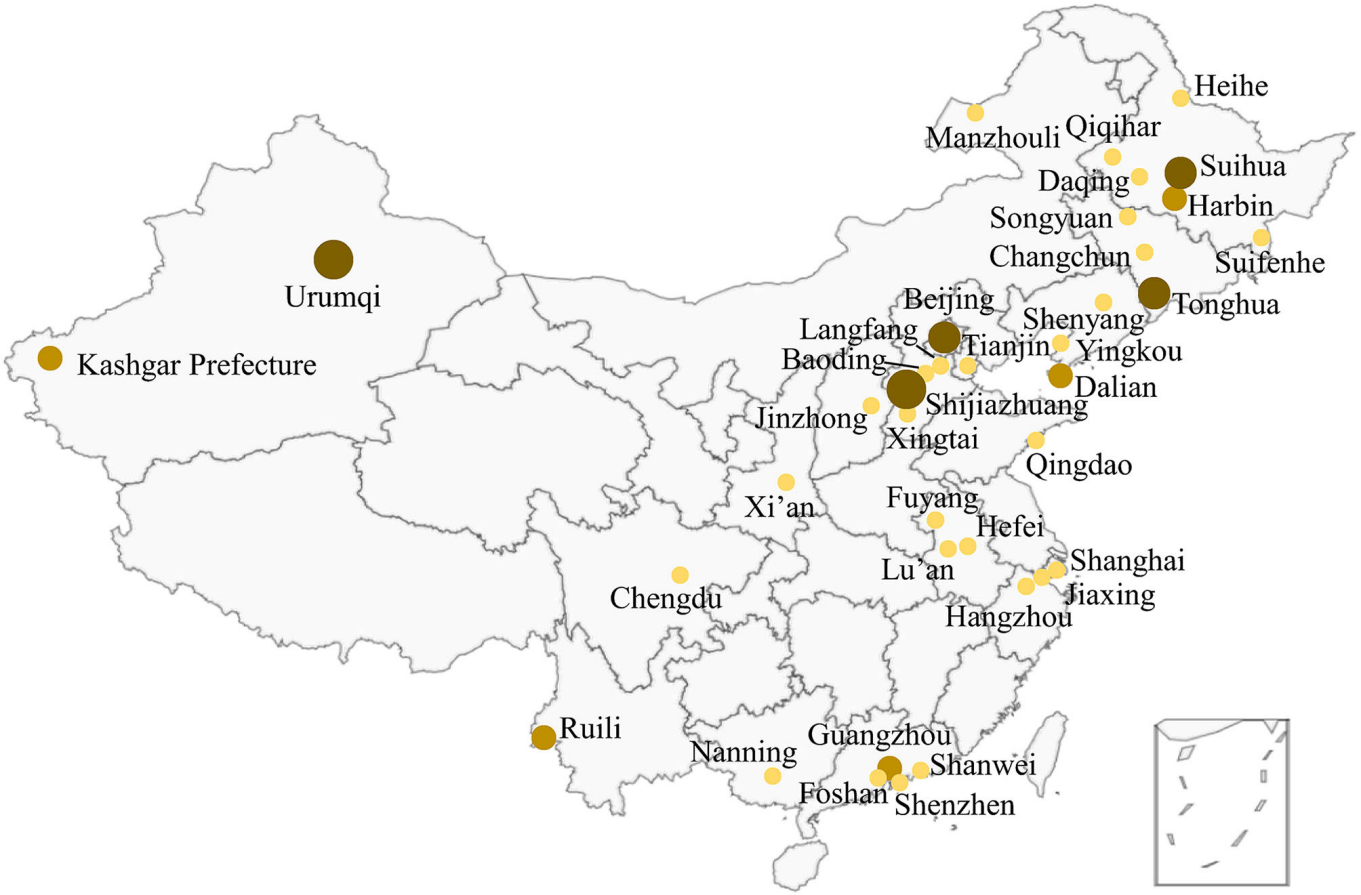
Location distribution of the outbreaks in Chinese mainland. There were two or more outbreaks in some cities.

By June 13, 2021, 43 new indigenous outbreaks have occurred in Chinese mainland, mainly distributed in central cities, border cities in the inland areas and coastal port cities (Figure 2). The cities can be divided into five categories, including central cities, such as Beijing, Shanghai, Tianjin, Guangzhou, Chengdu and Shenzhen; entry-exit cities during COVID-19 pandemic, such as Shijiazhuang, Harbin, Shenyang; inland border cities, such as Suifenhe, Heihe, Ruili, Manzhouli; coastal port cities, such as Dalian, Yingkou, Qingdao; other cities, such as Suihua, Tonghua, Lu'an and Xingtai. Meanwhile, the large-scale outbreaks were mainly occurred in the cities and seasons with low temperature.

### Infection Routes of First Generation Indigenous Patients

In the new indigenous outbreaks, the infection routes of the first generation patients were mainly direct or indirect contact with imported patients, goods or environments contaminated by SARS-CoV-2. Therefore, the indigenous patient included staff or nearby residents contacted with imported cases in quarantine hotels, families infected by imported cases during home quarantine, and staff contacted with contaminated imported cold chain food or environment of inbound aircraft and freighter.

### Infection Routes of Secondary Generation Indigenous Patients

The infection routes of secondary generation patients included family transmission, indoor social gathering infection (dinner party, wedding banquet, workplace, training class, market, etc.), nosocomial infection and other infection routes (Table 2). Family transmission was the most common infection route in secondary generation patients, which occurred in almost all the outbreaks. Indoor social gathering was the most important reason for the large-scale outbreaks. The outbreaks in Suihua and Shijiazhuang, including 1,359 symptomatic and 460 asymptomatic patients, were caused by wedding banquets. The transmissions of COVID-19 caused by dinner party, work place and training class were found in the outbreaks in Beijing, Harbin, Tonghua, Urumqi, and Guangzhou. COVID-19 transmission caused by nosocomial infection was also occurred in hospitals in some cities.

**Table 2:** Infection routes of outbreaks in five cities.

**City**	**Family transmission**	**Nosocomial infection**	**Indoor social gathering**	**Other**	**Total**
Harbin	14 (21.5)	46 (70.8)	4 (6.2)	1 (1.5)	65 (100.0)
Beijing	60 (17.9)	0 (0.0)	252 (75.2)	23 (6.9)	335 (100.0)
Dalian	19 (20.7)	0 (0.0)	31 (33.7)	42 (45.7)	92 (100.0)
Tonghua	46 (15.0)	0 (0.0)	107 (34.9)	154 (50.2)	307 (100.0)
Guangzhou	76 (54.7)	0 (0.0)	6 (4.3)	57 (41.0)	139 (100.0)

## Discussion

Prevention of COVID-19 pandemic is the top priority in the world. After the outbreaks have been controlled in some countries or areas, the normal work, life and foreign exchanges should be restored. Therefore, the focus of prevention in these countries was to prevent the resurgence of COVID-19 epidemic due to imported patients. In this study, we analyzed the epidemiological characteristics and infection routes of new indigenous outbreaks in Chinese mainland in recent 1 year after the pandemic in Wuhan was controlled. The results showed that the new indigenous outbreaks mainly occurred in central cities, border cities in the inland areas and coastal port cities. The main infection routes of first generation patients were direct or indirect contact with imported cases, imported goods or related environment contaminated by SARS-CoV-2. The infection routes of the secondary generation patients mainly included family transmission, indoor social gathering infection, nosocomial infection and other infection routes. Although these outbreaks have been controlled in a short time, there were still some gaps in community prevention and nosocomial infection control in new COVID-19 outbreaks.

### Prevention of First Generation Patients

The first generation patients were the source of COVID-19 resurgence and the focus of preventive strategies. The first generation patients of 15 outbreaks (34.9% of all 43 outbreaks) were related with imported patients, suggesting the necessity of stricter isolation management of entry personnel (21–25). The criterion for isolation release of entry personnel in Chinese mainland was adjusted from “14-day isolation in quarantine hotel and negative nucleic acid test” to “14-day isolation in quarantine hotel and negative nucleic acid test, followed by 7-day home quarantine and negative nucleic acid test.” Meanwhile, staff in quarantine hotel should keep strict personal protection, health monitoring and regular nucleic acid test according to the guideline for isolation of entry personnel (20). The quarantine hotel should be cleaned and disinfected, and all garbage should be disposed according to the treatment of medical waste. Home quarantine was commonly used in COVID-19 prevention, which was mainly suitable for people who have completed 14-day isolation in quarantine hotel. However, there were several cases of COVID-19 transmission in the period of home quarantine. Therefore, in addition to the current quarantine strategy, we proposed that the following stricter home quarantine standards should be taken: living alone, prohibition of going out, ventilation of the isolation room, regular disinfection and nucleic acid test. Further studies were needed to explore the relationship between the conditions of home quarantine and the risk of disease transmission, so as to avoid the disease transmission during home quarantine, especially the family transmission and the infection of neighbors.

The first generation patients of seven outbreaks (16.3% of all 43 outbreaks) were related with the contaminated imported goods or environments, including cold chain food, container, freighter, aircraft and so on (26–28). Therefore, the imported goods should be strictly sampled, detected, disinfected and traced according to management scheme (20). Meanwhile, in addition to personal protection, vaccination and regular nucleic acid test, we considered that the staff in high-risk posts of infection should be managed in close system, and the staff in low-risk posts should be managed by limiting going out, keeping strict personal protection, recording the travel track during going out and carrying out regular nucleic acid test for their families (29, 30).

### Prevention of Secondary Generation Patients

Compared with the first generation patients, the infection routes of secondary generation patients were more diversified, and there were a large number of asymptomatic patients, which increased the difficulty of prevention (31, 32). In the infection routes of the secondary generation patients, the transmission related to indoor social gathering was the key factor of the epidemic size. Large-scale indoor social gathering infection occurred in five outbreaks with the largest number of confirmed patients (Beijing, Shijiazhuang, Urumqi, Suihua, and Tonghua), four of which occurred in rural areas with weak medical condition (33–35). The control of these outbreaks proved that the current preventive strategies should be sufficient to control the transmission of COVID-19. Therefore, we emphasized that shopping mall, farmers' market, school, restaurant and other indoor public place still needed to strictly implement the prevention measures, including disinfection, ventilation, strengthening personnel health monitoring and protection, using COVID-19 screening application and controlling occupant density, even when there was no indigenous outbreaks during the worldwide COVID-19 pandemic. Meanwhile, epidemic investigation, cooperation with community medical organization and web-based trajectory can be used to accurately track close contacts. Especially for rural areas with weak personal protection awareness and medical condition, the policy of registration and health monitoring for returning personnel should be strengthened to detect the potential patients and close contacts (36).

Nucleic acid test was an important method for etiological diagnosis of COVID-19, especially for asymptomatic and atypical patients. However, false negative results were found in some imported patients, indigenous symptomatic and asymptomatic patients before diagnosis. In addition to the low viral load in some patients' nasopharynx, it may also be related with the process of collection and transportation of specimens (37, 38). Therefore, during the collection of nasopharyngeal and throat swabs, we suggested that the staff needed to follow the guideline for SARS-CoV-2 specimen collection and test, with the emphasis on that the samples should be collected from the tonsil and the posterior wall of nasopharyngeal cavity, the wipe times should be enough to collect valid specimens, and the specimens should be sent for inspection under cold chain condition as soon as possible.

In summary, the prevention of new indigenous epidemic in areas initially under control was an important issue during the pandemic of COVID-19. We analyzed the new indigenous outbreaks in Chinese mainland, and found that these outbreaks mainly occurred in central, border and coastal port cities. The first generation patients were mainly infected by contacting imported patients, contaminated goods and environment, and secondary patients were mainly infected through family transmission, indoor social gathering and nosocomial infection. Strengthen the management of imported patients and staff at high-risk of SARS-CoV-2 infection was the key point to avoid the first generation indigenous patients. When the new indigenous epidemic occurred, prompt management of indoor public place and monitoring of population at risk of infection using big data can avoid large-scale epidemic.

## Data Availability Statement

The raw data supporting the conclusions of this article will be made available by the authors, without undue reservation.

## Author Contributions

YL: investigation, visualization, and writing—original draft preparation. KY: investigation, data curation, and writing—reviewing and editing. SZ: investigation, methodology, and writing—reviewing and editing. LW: investigation, writing—reviewing and editing, and conceptualization. RC: conceptualization and writing—reviewing and editing. All authors contributed to the article and approved the submitted version.

## Funding

This work was supported by the Emergency Research Project of COVID-19 of Zhejiang University (grant number: 2020XGZX024).

## Conflict of Interest

The authors declare that the research was conducted in the absence of any commercial or financial relationships that could be construed as a potential conflict of interest.

## Publisher's Note

All claims expressed in this article are solely those of the authors and do not necessarily represent those of their affiliated organizations, or those of the publisher, the editors and the reviewers. Any product that may be evaluated in this article, or claim that may be made by its manufacturer, is not guaranteed or endorsed by the publisher.
